# A study on the relationship of sexual satisfaction and common contraceptive methods employed by the couples

**Published:** 2010

**Authors:** Zahra Mehdizadeh Toorzani, Roshanak Hasan Zahraei, Soheila Ehsanpour, Mahmood Nasiri, Shahla Shahidi, Bahram Soleimani

**Affiliations:** * School of Nursing and Midwifery, Karaj Branch, Islamic Azad University, Tehran, Iran; ** Department of Midwifery, School of Nursing and Midwifery, Isfahan University of Medical Sciences, Isfahan, Iran; *** Department of Psychiatric Nursing, School of Nursing and Midwifery, Isfahan University of Medical Sciences, Isfahan, Iran; **** Isfahan Province Mothers Health Center, Isfahan University of Medical Sciences, Isfahan, Iran; ***** Department of Statistics, School of Health, Isfahan University of Medical Sciences, Isfahan, Iran

**Keywords:** Sexual function, sexual satisfaction, contraceptive methods

## Abstract

**BACKGROUND:**

Sexual relationship is a basis for mental health and continuity of the healthy generation. Enjoying the healthy body and mind will cause the sexual relationships to run their normal course in life. One of the problems that couples are faced within their sexual relationships is the issue of employing family planning methods. Studies have revealed that contraceptive methods are in connection with the sexual function and health in different ways. This study was aimed to determine the mean and the relation of scores of sexual satisfaction of men and women with the common contraceptive methods.

**METHODS:**

This was a descriptive-correlative study. Samples included 280 individuals (140 couples) to use the common contraceptive methods including withdrawal method, tubal ligation in women, oral contraceptive method, condom, vasectomy, IUD and injection contraceptive method. Tools for gathering the data were Female Sexual Function Index (FSFI) and the questionnaire provided by Dr. Abdo on sexual satisfaction in men in 2004. The validity and reliability of these questionnaires were approved by researches conducted in worldwide and Iran. Descriptive and inferential statistic methods were applied in analyzing the data.

**RESULTS:**

The results suggested a significant statistic relation between scores of men’s sexual satisfaction and separate contraceptive methods (p = 0.001) whereas this relation was not observed between the women’s scores of sexual satisfaction and the contraceptive methods.

**CONCLUSIONS:**

According to the results of the present study, training family planning counselors in relation to choose suitable contraceptive method, in view of its probable effects on the couple’s sexual satisfaction, seems essential.

Family is one of the fundamental elements of the social system, and the family health will bring about a suitable environment for growth of spouses and their children. Principally, spouses’ relationships form on the basis of emotive, cognitive, economic, and sexual relationships. Any disorder in bilateral process of relationships can be the causation of problems and unsteadiness in the consolidation of family.^1^,^2^ A large number of researchers hold that sexual relationship is one of the most important causes of luckiness and unluckiness in the conjugal life, since such a relationship will result in feeling of inadequate deprivation and insecurity if it is not satisfactory. Healthy sexual relationships lead to achievement of affinity and affection in family in addition to proper satisfying of sexual instincts.^3^ Orgasm is one of the great divine blessings which relieves many inner tensions and brings about mental peace. This phenomenon is a normal ecstasy and stupefying which is similar to the water poured onto the fire of man’s inner agitations and excitements. The ability to express the sexual affairs positively is one of the most enjoyable and fruitful human aspects.^4^ Conjugal relations are among the social manners which, as history shows, have been common in all human societies to date, and this shows by itself that it is a natural, instinctive will.^5^ But one of the problems couples are faced within their sexual relationships is the issue of necessity of family planning and reducing the dramatic growth of population according to its special problems.^6^

World’s population increases 150 people per minute, 220’000 people per day and 80 million each year that 90 percent of these are related to the third world countries and their population is doubled every 37 years.^7^ So, the best solution in this global fight to reduce the population is to implement and expand family planning programmes.^8^ Family planning programs help women control number, interval, and the time of pregnancies and births by interfering in their cycles of fertility.^9^ According to statistics reported by the national research of integrated management and evaluation in Iran, 78.9 percent of people of Iran in 2005 used the contraceptive methods.^10^ Making decisions on the part of a couple on beginning to use a contraceptive method is influenced by the wishes, expectations, fears, and anxiety relating to the expected sexual activity in such a way that this anxiety exists in the person from the very beginning of selecting the method that whether the method will be positive and effective and will not have negative effects on the sexual relationships.^11^ Costello et al study showed that more than 80 percent of women under study did not report interest and sexual pleasure after sterilization.^12^ Also, Souki and Sharifi did not observe significant relationship between the use of condoms or other methods and changes of libido.^13^ There are no statistics on quitting contraception methods in Iran. Due to the importance of sexual issues in maintaining physical and mental health of individual and family as well as special cultural and religious conditions of Iran regarding sexual function and selecting contraceptive methods, this study was done with the purpose of determining the mean scores of sexual satisfaction in users of different types of common contraceptive methods and the relation between the scores and the methods adopted, thereby contributing to the quality of life of couples by improving their sexual satisfaction with the sexual relationships and finding appropriate counseling in this respect.

## Methods

This was a descriptive-correlation study. Samples consisted of 280 individuals (140 couples) who used common contraceptive methods based on the Integrated Management and Evaluation Study (IMES), carried out in the city of Ifahan, included coitus interruptus, tubal sterilization, oral contraceptive pills, condom, vasectomy, IUD, and injection contraceptive methods; 20 couples were equally chosen for each method. These subjects were simply selected from among those who referred to the health-treatment centers to receive such services. Health-treatment centers were selected through random cluster method.

Inclusion criteria for this study included all Iranian Muslim couples who had married for the first time, their marriage was of monogamy type, they were cohabiting, and at least 6 months had elapsed from their proper, continuous use of one of the methods. Women had to be within the range of age of fertility (15-45 years), and men had to have at least junior high school diploma. Moreover, all diseases or medicines which affected the sexual function of couples were questioned and if positive, they were excluded from the study. Besides, addiction of each couple, their growth in single-child families, record of sexual abuse, abortion or miscarriage during the past one year, experiencing severe stress during the last one year, serious family disputes in the last month, breastfeeding women, and having no child were among other criteria of being excluded from the study.

The time of gathering the data was from 2008 February to 2008 May. After obtaining permission from the units studied, necessary information were collected by means of questionnaires concerning individual-fertility characteristics and Female Sexual Function Index (FSFI) and by asking questions from women in a calm and private environment. Then, sexual satisfaction questionnaire, prepared by Dr. Abdo in 2004^14^ along with the written instructions of the guide to fill up the questionnaires were sent to their spouses in a closed envelope, and the further appointment was made for collecting the questionnaires. Questionnaire of individual characteristics included questions on relationship with husband and wife’s ages, level of husband and wife’s education, their jobs, recent contraceptive method, and duration of using last contraceptive method. Women’s sexual satisfaction was studied with answering to question numbers 14, 15 and 16 of FSFI questionnaire. Minimum and maximum scores obtained from the section of women’s sexual satisfaction were 0.8 and 6, respectively. Men’s sexual satisfaction was discussed through Dr. Abdo’s 10-item questionnaire, designed in 2004, and the calculated score multiplied by 2, resulting in minimum and maximum scores of 0 and 100, respectively. Validity and reliability of FSFI questionnaire and Dr. Abdo’s 2004 questionnaire on the sexual satisfaction were confirmed according to the researches carried out nationwide and worldwide.^14^,^15^

The results obtained from this study were analyzed with Pearson, Spearman and ANOVA tests, by the help of SPSS^11.5^ software. In this research, 5% error was designated.

The ethical committee of Isfahan University of Medical Sciences approved the study.

## Results

In terms of individual-fertility characteristics of the units studied, the average age of the studied women was 31.4 years, and that of men was 36.4 years; 52.2% of women and 46.6% of men with the highest frequency had high school diploma; 82.9% of women were housewives and 56.4% of men were freelancers.

The highest mean (SD) scores of sexual satisfaction in women was seen in those with vasectomy in their husbands, 4.94 (0.92), and those who used low-dose estrogen (LD) pills, 4.88 (1.22). However, the lowest average scores of sexual satisfaction in women were seen in those with tubal ligation method, 4.34 (1.04), and the withdrawal method, 4.48 (1.18). Also, the findings presented that the most common women’s sexual satisfaction scores (55.7 percent) was related to those average scores. ANOVA test revealed no significant relation between scores of women’s sexual satisfaction and common contraceptive methods (p = 0.56 and F = 0.81).

On the other hand, the highest mean (SD) scores of sexual satisfaction in men were seen in those with the injection contraceptive methods, 76.60 (12.70), and tubal ligation in women, 76.35 (10.35). However, the lowest average score of sexual satisfaction in men was seen with condoms, 62.60 (19.65). Also, the findings presented that the most common men’s sexual satisfaction scores (91.4 percent) was related to “well scores”. ANOVA test revealed significant relation between scores of men’s sexual satisfaction and common contraceptive methods (p = 0.001 and F = 4.54).

## Discussion

Results of the present study demonstrated significant relation between the mean scores of men’s sexual satisfaction and common contraceptive methods. However, there was no significant relation between the mean scores of women’s sexual satisfaction and common contraceptive methods. Results of Li et al study showed that scores of sexual satisfaction of women who had tubal sterilization were higher, three to four months after sterilization vs. other contraceptive methods, and these scores had a statistically significant difference from those of other methods.^16^ Results of Hasio et al study suggested no statistically significant relation between women’s sexual satisfaction and common contraceptive methods.^17^ Results of Ozgoli et al survey revealed that although scores of sexual satisfaction after tubectomy increased in the following three months, this difference was not statistically significant.^18^

The Salmani et al research revealed that among contraceptive methods, IUD had the lowest orgasm disorder and tubal ligation had highest orgasm disorder compared with other methods.^19^ According to Li et al study,^16^ sexual satisfaction of the sterilized women was considerably increased whereas in the present study, the mean score of sexual satisfaction of the sterilized women was among the lowest scores among different methods. Maybe one of the reasons for the difference is that in Li et al study, women’s sexual satisfaction was compared only three months after the operation with the period before the operation, while in the present study, average length of time elapsed from the sterilization operation was 6.5 years and women’s sexual satisfaction was not measured before the satisfaction. But, results of studies done by Hsiao et al^17^ and Ozgoli et al^18^ were similar to those reported in the present study. So, cultural conditions as well as lack of awareness of women in reaching the orgasm and satisfaction in their marital relationships lead to the results of this study. According to the research performed a few years ago in one of the cities in western Iran, more than 90 percent of urban married women in their lives never experienced orgasm.^20^ Results of the study done by Bertero et al demonstrated that sexual satisfaction of men undergoing vasectomy operation were significantly different three months after the operation compared with the time before the operation.^21^ Results of the study carried out by Hofmeyr et al indicated no significant difference in men’s sexual satisfaction and their satisfactions with the conjugal life before and five months after the vasectomy operation.^22^ It seems that general trainings for sexual issues are given to an extremely small extent on account of the cultural issues of the society; but men have more access to the sexual information than women due to a series of conditions and limitations, yet some of this information may have no scientific foundation.

Since one of the most important reasons for women’s sexual dissatisfaction is inadequate knowledge and experience in the field of sexual relationships,^23^ and women’s educational needs are more than men regarding the sexual relationships, the results of the present study can be interpreted.^24^ The results of this research can be acknowledged to the importance of proper training and consultation in connection with the sexual satisfaction and contraceptive methods, the cultural conditions of society and the importance of sexual satisfaction in marital life with emphasizing on contraceptive methods, providing the right information through the educational booklets as well as basic training in high school and university levels in this area seems necessary.

The authors declare no conflict of interest in this study.
